# Problem-solving in caregiver-counselling (PLiP Study): study protocol of a cluster randomized pragmatic trial

**DOI:** 10.1186/s12877-017-0456-x

**Published:** 2017-03-06

**Authors:** Klaus Pfeiffer, Martin Hautzinger, Margarete Patak, Julia Grünwald, Clemens Becker, Diana Albrecht

**Affiliations:** 1 Department of Clinical Gerontology and Rehabilitation, Robert-Bosch-Hospital, Auerbachstr. 110, 70376 Stuttgart, Germany; 2 Department of Psychology, Clinical Psychology and Psychotherapy, Eberhard Karls University, Schleichstr. 4, 72076 Tuebingen, Germany

**Keywords:** Caregiver, Counselling, Depression, Translational research, Problem-solving

## Abstract

**Background:**

Despite the positive evaluation of various caregiver interventions over the past 3 decades, only very few intervention protocols have been translated to delivery in service contexts. The purpose of this study is to train care counsellors of statutory long term care insurances in problem-solving and to evaluate this approach as an additional component in the statutory care counselling in Germany.

**Methods:**

A pragmatic cluster randomized controlled trial in which 38 sites with 58 care counsellors are randomly assigned to provide either routine counselling plus additional problem-solving for caregivers or routine counselling alone. The counsellor training comprises an initial 2-day training, a follow-up day after 4 months, and biweekly supervision contacts with a psychotherapist for 6 months over the phone. The agreed minimum counselling intensity is one initial face-to-face contact including a caregiver assessment and at least one telephone follow-up contact. Caregivers who are positively screened for significant strain in their role are followed up at 3 and 6 months after baseline assessment. Main outcome are caregivers’ depressive symptoms.

**Discussion:**

While it is unclear if the expected very low amount of additional counselling time is sufficient to yield any additional effects on caregiver depression, it is also unclear if the additional problem-solving component yields to synergies with routine counselling that is based on information and case management. There are different potential individual and organisational barriers to a consistent intervention delivery like gratification for participation, time for extra work or internal motivation to participate.

**Trial registration:**

(ISRCTN23635523)

## Background

Approximately 1.86 million (82.7% of them are ≥ 60 years old) out of 2.6 million care recipients in Germany are living at home. Within this group, the majority (1.25 million) receives support exclusively from informal caregivers without using any professional services [1]. Caregiver burden or distress is common [2] and associated with female gender, low education, residence with the care recipient, higher number of hours spent caregiving, depression, social isolation, financial stress, and the lack of choice being a caregiver [3]. Interventions to support informal caregivers have been evaluated in diverse disease specific contexts like dementia or stroke [4, 5]. Major components of interventions are psychoeducation, supportive interventions, psychotherapy, respite/day-care, or training of the care recipient [6]. The interventions are delivered in various ways (e.g. face-to-face or by telephone) and during different stages of the caregiving trajectory. Major endpoints e.g. in dementia caregiver research are depressive symptoms and emotional distress, burden, self-efficacy and coping, and quality of life [7]. Most of the evaluated heterogeneous interventions for dementia caregivers were effective to some extent [4], with a robust corpus of intervention studies that demonstrates small but clinically and statistically significant benefits for families [8]. Despite the evaluation of more than 200 dementia caregiver interventions over the past three decades, only very few programs have been translated to delivery in service contexts [9]. These rare efforts demonstrate the multiple challenges between required modifications according to staff and organization needs and the preservation of the integrity of the particular intervention [10].

Over the last years, the German legislature has initiated several new laws within the long-term care act to support care recipients and their caregivers. One regulation (social act / §7a SGB XI) that became effective in 2009 specifies the right for care recipients to get counselling and support by care counsellors (German: “Pflegeberater”). The counsellors usually have qualifications in the field of social work, nursing, or social insurance plus a defined specific training in nursing knowledge, case management, and legislation. The scope of counselling in this context should range from information transfer to case management. Care plans should be made in accordance with family caregivers who (and not the care recipient as insured person) is in most consultations the counsellor’s contact person. Recent laws and counselling practice do not consider caregivers as advice seekers with own needs and interests in a systematic way. While some long term care (LTC) insurances have explicitly addressed caregiver issues in their draft papers or even assessments, the current counselling practice is (to our knowledge) not directly linked to the corpus of evidence-based interventions in this field so far. The first evaluation report from 2012 summarizes that the professional qualification of care counsellors has made good progress, but the ways of implementation are very heterogeneous with regard to insurances, local districts, and states. Burden, mental or physical health outcomes of caregivers, as well as aspects of quality of care were not considered in this national evaluation [11].

From 2007 to 2010 we conducted a randomized controlled trial (ISRCTN86289718) to evaluate the impact of a mostly telephone-based problem-solving (PS) intervention to distressed family caregivers of German geriatric stroke survivors. The intervention’s effectiveness on depressive symptoms and physical complaints of the caregivers after the main intervention period (month 3) and the maintenance period (month 12) compared to a control group has significantly been proven [12]. Recent reviews on stroke [13] and dementia [14] caregiver research can underline positive effects of problem-solving interventions for this target group. The prevailing conceptualization of problem solving articulated by D’Zurilla and Nezu [15] describes PS as a general coping strategy with reciprocal relations to stressful life events and emotional stress responses.

The aim of the current study (2013 to 2016) is to train counsellors from nursing care insurances to apply problem-solving as a caregiver specific component and part of an extended care counselling. In a pragmatic trial we want to evaluate the effect of this extension compared to routine counselling on distressed caregivers. In line with the mixed samples of caregivers who receive counselling from LTC insurances, we want to include caregivers of care recipients with all kind of diagnoses (e.g. dementia, stroke, and heart failure). This is in contrast to most of the previous caregiver research that is segmented along different care recipient diagnoses and funding schemes. For the evaluation a cluster randomized design was chosen to prevent contamination of routine counselling of untrained counsellors by improved counselling skills of trained colleagues within the same office. Furthermore, we wanted to prevent that each participating counsellor would have had to switch between two different counselling approaches depending on the group allocation of the caregiver.

## Methods

### Design of the study

**Fig. 1:**
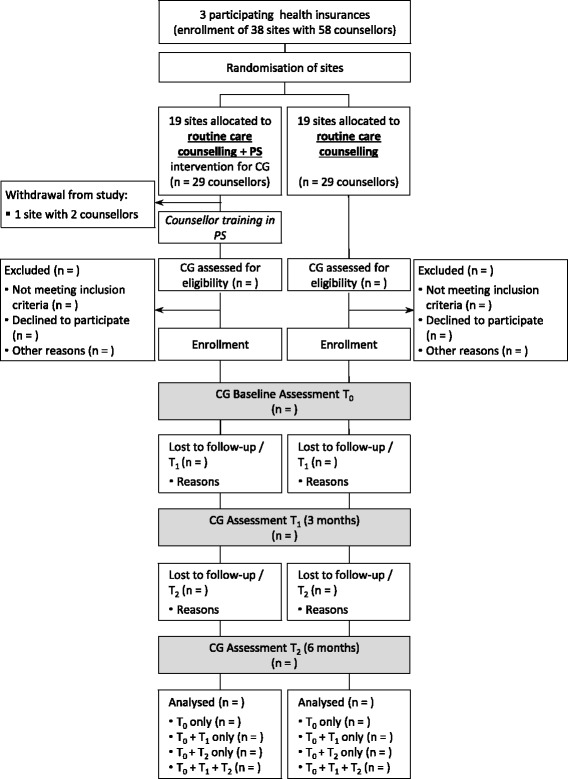
Recruitment and participation flow chart of care counsellors and caregivers

“Problem-solving in caregiver counselling” (German acronym: PLiP - ProblemLösen in der Pflegeberatung) is a translational study with a prospective cluster randomized design. Clusters are district offices of the participating nursing insurances with usually one care counsellor per office. But depending on the insurance and district size, some local offices with more than one counsellor are also included. The clusters were randomly assigned to the problem-solving training group and a control group (Fig. 1). The counsellors of the control group receive the training after finishing the evaluation. The allocation ratio was 1:1. We used a stratified block randomisation grouping district offices into strata defined by number of counsellors per office, and performing block randomisation within each stratum. The computer-generated, controlled random allocation of each local office is provided by an independent randomization centre at the University of Ulm and performed stepwise for each of the three participating insurances after entering the study.

The aim of this trial is to evaluate the impact of an advanced training in problem-solving for care counsellors on family caregivers who experience burden and depressive symptoms compared to usual counselling.

### Participants

#### Caregiver counsellors

Recruitment criteria on site of the caregiver counsellors are: 1) providing caregiver counselling in line with the German Social Security Code (§7a SGB XI), 2) qualification for counselling according to the recommendations of the National Association of Statutory Health Insurance Funds, 3) agreement to participate in the advanced training and evaluation of this study. Counsellors are excluded when their participation in the training course is incomplete (non-participation in the 2-day main training course, or/and less than 6 of the 13 bi-weekly supervision telephone contacts).

#### Informal caregivers

Inclusion criteria for informal caregivers are: 1) report distress associated with caregiving (endorsing at least two of three items asking if they feel their mental or physical health has declined due to caregiving tasks, feel lonely during the last week, or experience moderate to high levels of general stress), 2) comprehensive counselling or case management is necessary (at least one personal counselling session and at least one telephone-based or personal follow-up contact are planned), 3) having the primary responsibility for someone who is dependent on care according to the criteria of the German statutory nursing insurance, 4) is the main contact person for the care counsellor, 5) ≥18 years, and 6) consented to participate in the study. Exclusion criteria are: 1) professional paid responsibility for the care recipient, and 2) inability to speak and read German.

#### Recruitment of care counsellors and caregivers

Care counsellors are recruited from three German LTC insurances. The realization of the project is organized together with the person of each insurance who is responsible for care counselling. Depending on the specific LTC insurance, the participation of the counsellors within the project is either by choice or appointed by a principal. All counsellors do not receive extra money or time budgets for participating in the study.

Six informal caregivers should be recruited by each counsellor. For this purpose, caregivers who agreed should be screened consecutively by the counsellors for subjective caregiver burden. In case of a positive screening, the counsellor informs the caregiver about the basic facts of the study and asks for the caregiver’s consent to forward his contact details to the study evaluation team. The evaluation team informs about further details of the study to enable the caregiver to make an informed decision whether to participate in the study or not. The baseline assessment must be finished not later than 3 weeks after screening. Family caregivers receive either regular or advanced counselling by the participating counsellors. However, they are blinded and do not know whether their counsellor pertains to the intervention or control group.

### Intervention

The counsellors randomized to the intervention condition receive a specific training on PS that is based on the six principles of the PS model developed by D’Zurilla and colleagues [15–17]: (a) optimism and orientation, (b) problem definition and facts, (c) goal setting, (d) generation of alternatives, (e) decision making, and (f) implementation and verification. The training in PS in our study addresses three facets: (1) Facilitating the caregiver PS as the major component of the training. For problem identification, we trained the counsellors in using a card-sorting task with 37 cards covering possible (care related) problems. The used cards are based on previous research [12, 18, 19] and modified together with participating care counsellors for this study. As additional materials, three worksheets are provided to support the PS process (situation analyses based on the ABC model (A = adversity, B = beliefs, C = consequences [20]); goal definition, solution generation and evaluation; solution implementation and verification). (2) Using short presentations, small group discussions, and role-plays we apply the described PS steps to improve or overcome difficult interactions between counsellors and caregivers. (3) Other aspects of work-related difficulties or mental hygiene are mainly addressed during the individual telephone contacts. The training is delivered in an initial 2-day workshop, a follow-up day after 4 months, and individual bi-weekly telephone supervision contacts over 6 months after the initial workshop to facilitate the implementation of the PS principles and the card-sorting task in daily counselling practice.

While the on-site counsellor trainings in small groups (maximum 12 counsellors) are highly structured, the telephone contacts focus on implementing PS for caregivers in daily routine but also include a tailored educational component based on the counsellor’s individual needs and wishes. Possible topics are coping with stressors (e.g. role stress, coping with labor conditions, organization of work, coping with death and dying) and specific strategies and techniques (e.g. resource activation, work-life balance, self-distancing, self-awareness and mindfulness-based techniques). Each supervisor has a list with potential relevant stressors and techniques to facilitate this training component that was implemented not least because to offer the counsellors something they can benefit directly.

The two trainers and supervisors are cognitive behavioral therapists without specific previous experience in caregiver research or care counsellor training. Both are paid on fee basis. Together with at least one of the supervisors, the principal investigator of the study (KP) is involved in the group trainings and has regular meetings with both supervisors.

After the 6-month training the counsellor should use the PS approach including the card-sorting task as an additional component of the usual care counselling (§ 7a SGB XI) with distressed caregivers who are included in the study. The PS steps should be delivered in one initial face-to-face contact (including the card-sorting task) and at least one telephone follow-up contact. During the evaluation period over 6 months the counsellors have four additional contacts with their supervisor. Further contacts with the supervisors are possible if needed.

In addition to the specific counselling, participating caregivers in the intervention group receive five standard information letter (LTC insurance, relief of caregiver strain, depression, problem-solving, relaxation) and if desired up to five more specific letters (nutrition, coping with problem behavior, fall prevention, pain, oral hygiene) by post within 6 month.

### Control group

The caregivers who were recruited by the counsellors of the control group receive advice in the context of the statutory care counselling (§7a SGB XI).

All care recipients and caregivers of the intervention and control group are getting additional mandatory counselling visits (in line with the German Social Security Code § 37.3 SGB XI) when receiving only lump-sum transfers and no non-cash benefits like professional support paid by the LTC insurance.

### Evaluation and outcomes

The project is evaluated on two different levels: 1) impact of the extended care counselling on the informal caregivers, and the training 2) on the care counsellors.

### Primary outcome measure

The primary outcome measure is depressive symptoms of the informal caregiver assessed by the 20-item Centre for Epidemiological Studies Depression scale (CES-D) [21, 22] at T_0_ (after enrollment), T_1_ (3 months after T_0_), and T_2_ (6 months after T_0_). Total scores range from 0 to 60 with a score of ≥16 as an indicator of significant symptoms [23]. Table 1 provides an overview of the primary and secondary outcomes that will be measured at the three time points.

**Table 1:** Primary and secondary outcomes collected at the three time points

Measures	Tools	T_0_	T_1_	T_2_
Primary outcome (caregivers)				
Depressive symptoms	CES-D	x	x	x
Secondary outcomes (caregivers)				
Caregiver burden	SCQ (short version)	x	x	x
Subjective physical complaints	GBB-24 (subscale: Pains in Limbs)	x	x	x
Negative Problem Orientation	SPSI-R (subscale: Negative Problem Orientation)	x	x	x
Leisure Time Satisfaction	LTS	x	x	x
Secondary outcomes (care counsellors)		T*_0_	T*_1_	T*_2_
Session management self-efficacy	CASES (subscale: Session Management Self-Efficacy)	x	x	x
Difficult client behaviors self-efficacy	COSE (subscale: Difficult Client Behaviors)	x	x	x

Abbreviations: *CASES* counselor activity self-efficacy scales, *CES-D* centre for epidemiological studies depression scale, *COSE* counseling self-estimate inventory, *GBB-24* Giessen subjective complaints list, *LTS* leisure time satisfaction questionnaire, *SCQ* sense of competence questionnaire, *SPSI-R* social problem solving inventory - revised

*Measurement points: T*_*0*_ *= baseline, T*_*1*_ *= 3 months, T*_*2*_ = 6 months, T*_0_ , = basline, T*_1_ = 6 months, T*_2_ *= 12 months*

### Secondary outcome measures

#### Informal caregivers

Caregiver burden is assessed by a short version [24] of the Sense of Competence Questionnaire (SCQ) [25]. It contains 16 items that are rated on a five-point scale with higher scores indicating a greater sense of competence. Total scores range from 16 to 80. Subjective physical complaints are assessed by the Giessen Subjective Complaints List (GBB-24) - subscale ‘Pains in Limbs’ [26] that addresses six symptoms like headache, backache, sense of heaviness and fatigue in legs. The intensity of each of the six complaints is rated on a five-point scale, ranging from 0 (not existing) to 4 (strong). The subscale ‘Negative Problem Orientation’ of the Social Problem Solving Inventory – Revised (SPSI-R) is used to measure negative problem orientation [27, 28]. This subscale consists of five items that are rated on a five-point scale ranging from 0 (not very true of me) to 4 (extremely true of me). The total score ranges from 0 to 20. Besides, leisure time satisfaction is assessed by the Leisure Time Satisfaction Questionnaire (LTS) [29]. Items are rated on a Likert-type scale ranging from 0 (not at all) to 2 (a lot). The total score ranges from 0 to 12, and higher scores reflect greater satisfaction. In the validation study [29] the median of the LTS was 5 in a sample of 1,229 dementia caregivers (mean age = 62.3 years).

Further variables are the caregiver’s care-related quality of life (CarerQol) [30], informal care activities, and semi-structured qualitative interviews with a random sample of caregivers.

Additionally, some care recipient characteristics are assessed. Functional disability is measured with the Barthel Index of ADL (BI) [31], higher cognitive functioning (comprehension, verbal expression, social interaction, problem solving, memory/learning/orientation, and vision/neglect) with the Extended Barthel Index (EBI) [32]. Besides, retrospective data on health service use and the amount of formal and informal care are collected at each assessment for the previous 3 months.

All caregiver domains (except the qualitative interviews) are measured at T_0_ (after enrollment), T_1_ (3 months after T_0_), and T_2_ (6 months after T_0_) via telephone interview. Assessors are trained PhD students or research assistants who are blind to the treatment condition. Caregivers are provided with some of the questionnaires to make the interviews easier for them.

#### Care counsellors

As further secondary outcomes we assess counsellor’s self-efficacy with questions from the Counselor Activity Self-Efficacy Scales (CASES) - subscale ‘Session Management Self-Efficacy’ [33] and the Counselling Self-Estimate Inventory (COSE) - subscale ‘Difficult Client Behaviors’ [34]. Additional measures are the Maslach Burnout Inventory (MBI) with the subscales ‘Emotional Exhaustion’, ‘Depersonalization’, and ‘Personal Accomplishment’ [35, 36], a check-list for self-care [37], and the Trier Inventory of Chronic Stress (TICS) - subscale ‘Work Overload’ [38]. Furthermore, the quality of counselling is rated by the informal caregivers at T_1_ and T_2_.

Counsellors assigned to the intervention group are evaluated at T*_0_ (before the training), at T*_1_ after finishing the training and before the evaluation of their clients (6 months after T*_0_), and at T*_2_ (12 months after T*_0_). Counsellors assigned to control group are evaluated at T*_0_ (before evaluation of their clients), T*_1_ (before training: 6 months after T*_0_), T*_2_ (after the training: 12 months after T*_0_). The counsellors receive the paper-and-pencil questionnaires with their code number by post.

### Sample size calculation

In a first step we conducted an a priori power analysis based on procedures from Borm, Fransen, and Lemmens [39] for analysis of covariance in randomized clinical trials. To detect an expected minimum effect size of .40 (Cohen’s d) after the 12-month period with power β = .80 (two-sided test, α = .05), assuming a correlation ρ = .70 between baseline and follow-up assessments, 52 participants in each group are needed. Because of the reduced power within a cluster-randomized design the number of participants (*n* = 104) was multiplied with a design factor (DE), that was calculated with the formula: DE = 1 + ICC*(m-1) (DE = Design effect, ICC = intra cluster correlation coefficient, m = number of caregivers per cluster). Each counsellor represents a cluster who recruits six caregivers (m = 6). The estimation of the ICC (ICC = 0.138) was based on previous data (Pfeiffer et al., [12]). By multiplication of the design effect by the calculated sample size without cluster effect (*n* = 104), a sample size of 88 caregivers in each group was obtained. Because we assumed that not all counsellors will recruit 6 caregivers, the target number for participating counsellors was 44 (20*6 + 10*4 + 8*2 + 6*0, while the first factors represents the “number of counsellors”, and the second factors the assumed “number of caregivers recruited by each counsellor”).

### Statistical methods

The comparability of the care counsellors and caregivers in the intervention and control groups are analyzed with independent samples t-tests on continuous variables, Mann-Whitney U on ordinal or continuous, but not normally distributed variables, and χ^2^ tests or Fisher’s exact tests of independence for categorical variables. Because of the fact that in this cluster RCT the number of randomized local offices and care counsellors is much smaller than the number of participating caregivers who are the unit of analyses we assume an increased likelihood of imbalance between caregivers in the intervention and control conditions on baseline characteristics. Such possible imbalances at baseline threaten the validity of inferences regarding intervention effects unless an appropriate statistical adjustment is used [40]. Therefore counsellors and caregivers are matched on propensity scores using the optimal matching approach before conducting treatment effectiveness analyses. Subsequent to the matching we compare the original sample with the matched sample. Treatment effects are tested with confirmatory endpoint analyses (analysis of covariance/ANCOVA; two sided 5% level) after three (T_2_) and 6 months (T_3_), using baseline scores as a covariate. For the intend-to-treat (ITT) analysis we use maximum-likelihood multiple imputation to impute missing values for withdrawn subjects or participants with missing data at month 3 and 6. The continuous longitudinal outcome data will be analyzed by random coefficient models to apply on the patient-specific time series, and, for purposes of confirmation, by an ANOVA with repeated measures. Mean effects per care counselor are compared between intervention and control condition.

## Discussion

The aim of this approach is to train caregiver counsellors to address specifically caregiver issues in addition to the recent care counselling routine. In our translation approach together with three LTC insurances with more than 9 million members we evaluate the effectiveness of a modified previously proven intervention approach [12] in this routine setting. As in previously published intervention studies [9], modifications have to be made to simplify our original intervention protocol to fit the delivery environment and resources of the LTC insurances. We could define with the participating LTC insurances only a minimum intensity for the additional counselling component that is well below the intensity in the successful efficacy trial [12]. It is unclear if this minimum dosage is sufficient to yield any effects on caregiver depression, while at the same time we do not know if the combination of our intervention component with the already existing statutory care counselling yields to synergies. Further crucial issues could be the counsellors’ adherence to the defined screening schedule and the proper delivery of the intervention. Possible moderators for the adherence could be lacking time for extra work, lacking gratification, but also the circumstance that some counsellors only participate in the study due to their supervisors’ directive. While all participating counsellors have a comparable specific training, the influence of their different original professions as social workers, nurses or social insurance employees on the delivery of the intervention is unclear. It is also possible that the counsellors of the control condition will provide a better counselling when they know they get evaluated. Furthermore, new laws and regular directives within the LTC insurances during the study might influence routine procedures as well as the workload of the participating counsellors.

## Abbreviations

ANOVA: Analyses of variance
CG: Caregiver
ICC: Intra cluster correlation coefficient
ISRCTN: International Standard Randomised Controlled Trial Number
LTC: Long term care
m: Number of caregivers per cluster
n: Number of participants
PLiP: ProblemLösen in der Pflegeberatung [Problem-solving in caregiver-counselling]
PS: Problem-solving
RCT: Randomized controlled trial
ref.: Reference
SGB: Social security code
T_0_, T_1,_ T_2_: Measurement points, T_0_ = baseline, T_1_ = 3 months follow-up, T_2_ = 6 months follow-up
